# Factors associated with undernutrition among 20 to 49 year old women in Uganda: a secondary analysis of the Uganda demographic health survey 2016

**DOI:** 10.1186/s12889-020-09775-2

**Published:** 2020-11-03

**Authors:** Quraish Sserwanja, David Mukunya, Theogene Habumugisha, Linet M. Mutisya, Robert Tuke, Emmanuel Olal

**Affiliations:** 1 Monitoring and Evaluation Department, Doctors with Africa, Juba, South Sudan; 2 Department of Research, Sanyu Africa Research Institute, Mbale, Uganda; 3 Centre for International Health, University of Bergen, Bergen, Norway; 4 Department of Women and Children’s Health, Uppsala University, Uppsala, Sweden; 5 Department of Psychiatry, Michigan State University, East Lansing, USA; 6 Yotkom Medical Centre, Kitgum, Uganda

**Keywords:** Under nutrition, Prevalence, Stunting, Underweight, Socio-economic, Women and Uganda

## Abstract

**Background:**

Women are at risk of undernutrition due to biological, socio-economic, and cultural factors. Undernourished women have higher risk of poor obstetric outcomes. We aimed to determine the prevalence and factors associated with undernutrition among women of reproductive age in Uganda.

**Methods:**

We used Uganda Demographic and Health Survey (UDHS) 2016 data of 4640 women aged 20 to 49 years excluding pregnant and post-menopausal women. Multistage stratified sampling was used to select study participants and data were collected using validated questionnaires. We used multivariable logistic regression to determine factors associated with underweight and stunting among 20 to 49 year old women in Uganda.

**Results:**

The prevalence of underweight and stunting were 6.9% (318/4640) and 1.3% (58/4640) respectively. Women who belonged to the poorest wealth quintile (Adjusted Odds Ratio (AOR) 3.60, 95% CI 1.85–7.00) were more likely to be underweight compared to those who belonged to the richest wealth quintile. Women residing in rural areas were less likely to be underweight (AOR 0.63, 95%CI 0.41–0.96) compared to women in urban areas. Women in Western (AOR 0.30, 95% CI 0.20–0.44), Eastern (AOR 0.42, 95% CI 0.28–0.63) and Central regions (AOR 0.42, 95% CI 0.25–0.72) were less likely to be underweight compared to those in the Northern region. Women belonging to Central (AOR 4.37, 95% CI 1.44–13.20) and Western (AOR 4.77, 95% CI 1.28–17.78) regions were more likely to be stunted compared to those in the Northern region.

**Conclusion:**

The present study showed wealth index, place of residence and region to be associated with undernutrition among 20 to 49 year old women in Uganda. There is need to address socio-economic determinants of maternal undernutrition mainly poverty and regional inequalities.

**Supplementary Information:**

The online version contains supplementary material available at 10.1186/s12889-020-09775-2.

## Background

Nutrition is a fundamental pillar of human life, health, and development [1]. Globally, 10% of women aged 20 to 49 suffer from underweight [2]. The greatest burden of maternal undernutrition is seen in low income countries [3] where an estimated 450 million adult women are stunted [4].

Women have a higher risk of undernutrition compared to men due to biological and socio-economic factors [2, 4, 5]. Particularly, negative gender norms that favor men over women such as men being served food first and women eating leftovers [6], and women not inheriting property are common in developing countries like Uganda [7, 8]. These norms lead to women having a lower socio-economic status compared to men [9] and disproportionately affected by under nutrition [10–12].

Undernutrition has far reaching consequences in women of reproductive age [13]. These consequences are experienced at the individual, community, and national level [14]. At the individual level, maternal undernutrition is associated with poor obstetric outcomes such as increased risk of maternal mortality and morbidity, preterm birth, low birth weight, still births, and increased risk of neonatal mortality [15]. Undernutrition reduces economic productivity through reduced labor productivity, high treatment costs, reduced wages and human capital losses [16–18]. This negatively affects community and national development through reduced family income and gross domestic product [16, 18]. Undernourished women are more likely to give birth to newborns with low birth weight and these newborns are at high risk of malnutrition leading to an intergenerational cycle of malnutrition [19].

Improving women’s nutrition is one of the ways of reducing undernutrition in children [3] and a strong pillar in the global efforts of reducing maternal mortality [20]. Uganda’s commitment to nutrition is ranked low [21]. Focus has been put on under 5 children and little research is available among women of reproductive age. In order to design interventions that address female undernutrition, estimating the burden and understanding potential determinants is critical. Therefore, in this nationally representative study, we aim to determine the prevalence and factors associated with undernutrition among women aged 20 to 49 years in Uganda.

## Methods

### Study design

We conducted a secondary data analysis of the 2016 Uganda demographic health survey (UDHS) data set.

### Data collection

This data were collected from 20th June 2016 to 16th December 2016 [11]. It was a nationally representative survey carried out by the Uganda Bureau of Statistics as part of the international MEASURE Demographic Health Surveys (DHS) with the support of ICF International and United States Agency for International Development (USAID). UDHS is a periodical survey conducted every 5 years. UDHS 2016 had four different questionnaires. Household questionnaire collected data on household environment, assets and basic demographic information of household members while women’s questionnaire collected data about women’s background characteristics, reproductive health, domestic violence and nutrition. The men’s questionnaire collected men’s health indicators’ data while the biomarker questionnaire collected data on anthropometry and blood tests [11]. Regarding anthropometry, weight was recorded in kilograms to the nearest one decimal point and was measured using an electronic scale (SECA 878) [11]. Height was recorded in centimeters to one decimal point.

### Study setting

As of July 2018, Uganda had a population of 40,853,749 million people with 23.8% of the population residing in urban areas [22] and the country has a total area of 241,551 km^2^ [23]. Uganda’s health system has six levels ranging from the highest level of national referral hospitals to the lowest level at the community level [24]. Agriculture contributes about 24% of gross domestic product (GDP), providing half of export earnings and is the main source of income for the 84% of Ugandans living in rural areas [25].

### Study sampling and participants

Samples were collected using stratified two stage cluster sampling design with census enumeration areas as the primary sampling units [11]. The first stage of sampling involved selecting 697 enumeration areas (EAs) including 162 urban and 535 rural enumeration areas selected from the list of the 2014 population and housing census sample frame [11]. One enumeration area in Acholi region was excluded due to land disputes hence ending up with 696 EAs. Enumeration areas with over 300 households were segmented and only one segment selected with probability proportional to the segment size as this helped minimize the burden of household listing [11]. The enumeration areas that were involved in the survey were chosen independently from each stratum with probability proportional to size. The second stage of sampling involved selection of households through equal probability systematic sampling. A list containing all households and maps in the selected enumeration area were made available and households that were in institutional living arrangements were excluded [11]. A power allocation with a small adjustment was done in the allocation of sample enumeration areas to ensure that the minimum number of clusters per survey domain are achieved. Finally, a representative sample of 20,880 households (30 per EA or EA segment) was randomly selected.

Women aged 15 to 49 years who were either the permanent residents or slept in the selected household the night before were eligible for inclusion in the Uganda’s demographic health survey 2016 [11]. Anthropometric measurements were done by trained technicians for about a third of the UDHS 2016 survey sampled women. The women sampled were neither pregnant nor had a birth 2 months before the survey [26]. Our secondary analysis only considered women aged 20 to 49 years and excluded women aged 15 to 19 years (adolescents) because the recommended anthropometric indicators (BMI and Height) for assessing undernutrition for those above 20 are different from those of adolescents (BMI for age and Height for age) and cannot be directly compared [27]. According to World Health Organization guidelines, measurement of adolescent nutritional status should account for age, as it is not until 19 years old that these curves approach convergence with adult models [27, 28]. According to the UDHS report, 18,506 women consented and filled in the questionnaires, of which 14,242 were aged 20 to 49 years [26]. Of these, 4731 were sampled for anthropometry and 4640 had their anthropometry done while 91 were not present or refused anthropometry to be done (Figure 1 in the supplementary file). We compared the socio-demographic characteristics of the women sampled for anthropometry to the socio-demographic characteristics of all women aged 20 to 49 years and there were no significant differences (Table 5 in the supplementary file). To maintain the representativeness of the sample and possible differences in response rates across regions, sampling weights were used.

### Outcome variables

In this study, underweight and stunting were the outcome variables. Underweight was defined as body mass index (BMI) less than (<) 18.5 kg/m^2^ while stunting was defined as height less than (<) 145 cm [3, 11, 20]. Underweight is further classified as severe (BMI less than 16.00 kg/m^2^), moderate (BMI 16.0016.99 kg/m^2^), and mild (BMI 17.00–18.49 kg/m^2^).

### Explanatory variables

This study included determinants of undernutrition basing on evidence from available literature and data [15, 20, 26, 29, 30]. These factors were divided into individual level (age, marital status, working status and education level), household level (wealth index, household size and sex of household head) and community level (region and residence) characteristics. Wealth index is a measure of relative household economic status and was calculated by DHS from information on household asset ownership using Principal Component Analysis [11, 31]. Different household assets were used to calculate separate wealth indices for rural and urban areas, combined into a national wealth index and these quintiles are; the poorest, the poorer, the middle, the richer and the richest quintiles [11, 31]. Place of Residence was aggregated as urban and rural. Region was categorized into four; Northern (Teso, Karamoja, Lango, Acholi, West Nile), Central (Kampala, Central 1 and Central 2), Eastern (Busoga, Bugishu and Bukedi) and Western (Tooro, Ankole, Bunyoro and Kigezi) [32]. Level of Education was categorized into: no education, primary education, secondary and higher education. Age was categorized into 20–29, 30–39 and 40–49. Household Size was categorized as less than six members and six and above members. Sex of Household Head was categorized as male or female. Working status was categorized as: not working and working. Marital Status was categorized into married and this included those in formal and informal unions and not married.

### Statistical analysis

The SPSS analytic software version 24.0 Complex Samples package was used for this analysis. Use of the complex samples package accounted for the complex survey sampling while use of sample weighted data accounted for the unequal probability sampling in different strata. Analyses were done by descriptive statistics and logistic regressions. Frequency tables and proportions/percentages were used to describe categorical variables while means and standard deviations for continuous variables. Initially, each exposure was assessed separately for its association with the outcome variables (stunting and underweight) using bivariate logistic regression and we present crude odds ratio (COR), 95% confidence interval (CI) and *p*-values. Independent variables found significant at *p*-value less than 0.2 [33–35] were included in the multivariable model. The non-significant variables whose associations with undernutrition had been shown in previous studies were also included in the multivariable logistic regression models.

In the multivariable analysis, we constructed two models based on the categorization of the independent variables into women individual and household and community level factors. We first performed a logistic regression model which included only individual characteristics (age, education level, working status and marital status). Then after, we constructed a final model which included individual characteristics adjusted for household and community characteristics (wealth index, residence, region, household size and sex of household head). Adjusted odds ratios (AOR), 95% Confidence Intervals (CI) and *p*-values were calculated with statistical significance level set at *p*-value < 0.05. Sensitivity analysis was done with only women who were underweight and had normal BMI after excluding those with BMI above 25.

## Results

A total of 4640 women were included in this study (Table 1). Over half of the women resided in rural areas (73.6%), were currently working (84.3%) and married (73.4%). In addition, more women lived in households with less than six members (53.9%), had primary education as the highest level (55.9%) and resided in male headed households (64.5%). Regarding geographical location, central region had the highest proportion of women (30.2%) while Eastern had the lowest (19.7%). Study participants are almost equally distributed with 20% in each quintile. The mean age, weight, height, household size and BMI were 31+ 8.18, 59.5 + 11.86, 158.9 + 6.37, 5.7 + 00, and 23.56 + 4.45 respectively.

**Table 1:** Background characteristics of Ugandan women aged 20 to 49 years as per the 2016 UDHS

Characteristics	***N*** = 4640	%
Age		
20 to 29	2225	48.0
30 to 39	1486	32.0
40 to 49	928	20.0
**Residence**		
Urban	1223	26.4
Rural	3416	73.6
**Region**		
Western	1182	25.5
Eastern	913	19.7
Central	1400	30.2
Northern	1144	24.7
**Sex household head**		
Female	1648	35.5
Male	2991	64.5
**Household Size**		
6 and Above	2138	46.1
Less than 6	2501	53.9
**Working status**^**a**^		
Not working	721	15.6
Working	3913	84.4
**Marital status**		
Married	3406	73.4
Not married	1234	26.6
**Education Level**		
No Education	555	12.0
Primary Education	2593	55.9
Secondary Education	1085	23.4
Higher	407	08.7
**Wealth Index**		
Poorest	816	17.6
Poorer	815	17.6
Middle	871	18.8
Richer	943	20.3
Richest	1195	25.7
**Underweight**		
Yes	318	06.9
No	4322	93.1
**Stunting**		
Yes	58	01.3
No	4581	98.7

^a^ = Working status had 6 missing values which was 0.1%

The prevalence of underweight and stunting were 6.9 and 1.3% respectively. Of those who were underweight, 77.4% were mildly underweight, 18.2% moderately underweight and 4.4% severely underweight. Distribution of undernutrition by background characteristics is shown in Table 2.

**Table 2:** Distribution of undernutrition by background characteristics among Ugandan women aged 20 to 49 years

Characteristics	Underweight	Not underweight	***P***-Value	stunted	Not stunted	***P***-Value
**Household Head**			0.166*			0.320
Female	124 (39.1)	1524 (35.3)		17 (29.3)	1631 (35.6)	
Male	193 (60.9)	2798 (64.7)		41 (70.7)	2950 (64.4)	
**Wealth Index**			< 0.001**			0.361
Poorest	114 (36)	701 (16.2)		8 (13.6)	808 (17.6)	
Poorer	74 (23.3)	741 (17.1)		10 (16.9)	805 (17.6)	
Middle	54 (17)	817 (18.9)		16 (27.1)	855 (18.7)	
Richer	39 (12.3)	903 (20.9)		8 (13.6)	935 (20.4)	
Richest	36 (11.4)	1159 (26.8)		17 (28.8)	1178 (25.7)	
**Working Status**^**a**^			0.299			0.747
Working	275 (86.5)	3638 (84.3)		49 (86)	3864 (84.4)	
Not Working	43 (13.5)	678 (15.7)		8 (14)	714 (15.6)	
**Education Level**			< 0.001**			0.210
No Education	62 (19.6)	492 (11.4)		9 (15.5)	546 (11.9)	
Primary	194 (61.1)	2399 (55.5)		33 (56.9)	2560 (55.9)	
Secondary	49 (15.5)	1036 (24)		6 (10.3)	1079 (23.5)	
Higher	12 (3.8)	394 (9.1)		10 (17.2)	397 (8.7)	
**Region**			< 0.001**			0.008**
Western	47 (14.8)	1135 (26.3)		21 (36.2)	1161 (25.3)	
Eastern	49 (15.5)	864 (20.0)		7 (12.1)	907 (19.8)	
Central	57 (18)	1344 (31.1)		24 (41.4)	1376 (30)	
Northern	164 (51.7)	979 (22.7)		6 (10.3)	1138 (24.8)	
**Marital Status**			0.398			0.201
Married	227 (71.4)	3179 (73.6)		39 (66.1)	3367 (73.5)	
Not Married	91 (28.6)	1143 (26.4)		20 (33.9)	1214 (26.5)	
**Age**			0.111*			0.150*
20 to 29	143 (45)	2083 (48.2)		35 (60.3)	2190 (47.8)	
30 to 39	97 (30.5)	1389 (32.1)		13 (22.4)	1473 (32.2)	
40 to 49	78 (24.5)	850 (19.7)		10 (17.3)	918 (20.0)	
**Residence**			0.013**			0.896
Rural	253 (79.6)	3163 (73.2)		43 (72.9)	3374 (73.6)	
Urban	65 (20.4)	1158 (26.8)		16 (27.1)	1208 (26.4)	
**Household Size**			0.027**			0.846
Six and Above	165 (52.1)	1973 (45.7)		26 (44.8)	2112 (46.1)	
Less than 6	152 (47.9)	2349 (54.3)		32 (55.2)	2469 (53.9)	

^a^ = Working status had 6 missing values which was 0.1%. ** = Significant at *p*-value < 0.05, * = Significant at *p*-value < 0.2 *

### Factors associated with underweight

After adjusting for individual characteristics, only education level was found to be associated with underweight. In the final logistic regression model, factors associated with underweight were: region, wealth index and residence (Table 3). Women residing in rural areas were 37% less likely to be underweight compared to those in urban areas (AOR = 0.63; 95% CI: 0.41–0.96). Women in the Western (AOR = 0.30; 95% CI: 0.20–0.44), Eastern (AOR = 0.42; 95% CI: 0.28–0.63) and Central (AOR = 0.42; 95% CI: 0.25–0.72) regions were 70, 58 and 58% less likely respectively to be underweight compared to those in the Northern region. Women belonging to the poorest (AOR = 3.60; 95% CI: 1.85–7.00), poorer (AOR = 3.07; 95% CI: 1.57–5.97) and middle (AOR = 2.49; 95% CI: 1.25–4.99) wealth index quintiles were 260, 207 and 149% more likely to be underweight compared to those in the richest wealth index quintile.

**Table 3:** Predictors of underweight among Ugandan women aged 20–49 years

Characteristics	Crude model (***n*** = 4640) OR (95%CI)	***P***-value	Model 1^**a**^ (***n*** = 4640) OR 1 (95%CI)	Sensitivity Model 2^**b**^ (***n*** = 3327) OR 2 (95%CI)
**Age**		0.131		
40 to 49	1		1	1
30 to 39	0.76 (0.54–1.06)		0.83 (0.59–1.17)	0.78 (0.55–1.10)
20 to 29	0.74 (0.55–1.00)		0.92 (0.66–1.28)	0.76 (0.54–1.07)
**Education Level**		< 0.001		
Higher	1		1	1
Secondary	1.50 (0.66–3.43)		1.43 (0.63–3.23)	1.29 (0.56–2.96)
Primary	**2.56 (1.17–5.57)**		1.58 (0.75–3.30)	1.42 (0.66–3.07)
No Education	**4.00 (1.77–9.07)**		2.16 (0.97–4.81)	1.96 (0.85–4.50)
**Marital Status**		0.409		
Not married	1		1	1
Married	0.89 (0.68–1.17)		0.81 (0.60–1.11)	0.89 (0.65–1.22)
**Working Status**		0.334		
Working	1		1	1
Not working	0.84 (0.59–1.20)		1.09 (0.75–1.58)	1.06 (0.73–1.54)
**Region**		< 0.001		
Northern	1		1	**1**
Western	**0.25 (0.18–0.35)**		0.30 (0.20–0.44) *	**0.35 (0.24–0.51)**
Eastern	**0.34 (0.23–0.50)**		0.42 (0.28–0.63) *	**0.44 (0.29–0.67)**
Central	**0.25 (0.16–0.39)**		0.42 (0.25–0.72) *	**0.51 (0.30–0.89)**
**Household Size**		0.04		
Less than 6	1		1	1
Six and above	**1.29 (1.01–1.65)**		1.13 (0.88–1.46)	1.13 (0.87–1.45)
**Wealth Index**		< 0.001		
Richest	1		1	1
Richer	1.41 (0.80–2.49)		1.56 (0.86–2.83)	1.20 (0.63–2.27)
Middle	**2.15 (1.24–3.71)**		2.49 (1.25–4.99) *	1.75 (0.85–3.62)
Poorer	**3.24 (1.92–5.46)**		3.07 (1.57–5.97) *	2.00 (0.99–4.01)
Poorest	**5.28 (3.25–8.57)**		3.60 (1.85–7.00) *	2.24 (1.12–4.48)
**Residence**		0.05		
Urban	1		1	**1**
Rural	1.43 (0.99–2.04)		0.63 (0.41–0.96) *	0.60 (0.39–0.94) *
Male	1		1	1
Female	1.18 (0.91–1.54)		1.16 (0.85–1.60)	1.15 (0.83–1.59)

* = Significant at *p*-value < 0.05, Final model - Adjusted for residence, region, sex of household, household size, occupation, marital status, age, education level and wealth index. Sensitivity model – Included only women with low and normal BMI and excluded women with BMI above 25.0. *AOR* Adjusted odds ratio. *COR* Crude Odds Ratio

### Factors associated with stunting

After adjusting for women individual characteristics, none of the individual characteristics were significantly associated with stunting. In the final model, only region was significantly associated with stunting (Table 4). Women belonging to the central (AOR = 4.77; 95% CI: 1.28–17.78) and Western (AOR = 4.37; 95% CI: 1.44–13.20) regions were 377 and 337% respectively more likely to be stunted compared to those in the Northern region.

**Table 4:** Predictors of stunting among Ugandan women aged 20–49 years

Characteristics	Crude Model OR (95%CI)	***P***-value	Final Model OR1 (95%CI)
Age		0.213	
40 to 49	1		1
30 to 39	0.78 (0.33–1.81)		0.67 (0.27–1.65)
20 to 29	1.47 (0.66–3.28)		1.51 (0.67–3.39)
**Education Level**		0.186	
Higher	1		1
Secondary	0.22 (0.05–0.94)		0.21 (0.05–0.88)
Primary	0.51 (0.16–1.61)		0.54 (0.17–1.68)
No Education	0.61 (0.16–2.36)		0.60 (0.16–2.24)
**Marital Status**		0.709	
Not married	1		1
Married	0.71 (0.37–1.37)		0.71 (0.28–1.79)
**Working Status**		0.732	
Working	1		1
Not working	0.85 (0.35–2.12)		0.71 (0.28–1.79)
**Region**		0.028	
Northern	1		1
Western	**3.63 (1.34–9.77)**		4.37 (1.44–13.20) *
Eastern	1.48 (0.43–5.13)		1.75 (0.46–6.71)
Central	**3.54 (1.25–10.00)**		4.77 (1.28–17.78) *
**Household Size**		0.825	
Less than 6	1		1
Six and above	0.93 (0.49–1.76)		1.04 (0.54–2.04)
**Wealth Index**		0.486	
Richest	1		1
Richer	0.60 (0.21–1.71)		0.69 (0.23–2.04)
Middle	1.31 (0.50–3.42)		1.33 (0.43–4.12)
Poorer	0.89 (0.33–2.41)		1.17 (0.36–3.75)
Poorest	0.70 (0.27–1.80)		1.35 (0.38–4.83)
**Residence**		0.962	
Urban	1		1
Rural	0.98 (0.44–2.20)		1.34 (0.57–3.16)
**Sex of Household Head**		0.410	
Male	1		1
Female	0.75 (0.37–1.50)		0.59 (0.31–1.14)

* = Significant at *p*-value < 0.05. Final Model - Adjusted for residence, region, sex of household, household size, occupation, marital status, age, education level and wealth index

### Sensitivity analysis

#### Analyzing after excluding obese and overweight women and considering only women with BMI < 25

We conducted a sensitivity analysis where we excluded women who had BMI above 25.0. The outcome underweight was divided into underweight (BMI < 18.5) coded as one and normal BMI (18.5–25.0) coded as zero. The final adjusted model showed association with only residence and region. Women residing in rural areas (AOR = 0.60; 95% CI: 0.39–0.94) were 40% less likely to be underweight compared to the urban ones and almost the same as those in the original analysis. Wealth index here unlike in the original analysis was not associated with underweight.

## Discussion

This study investigated the prevalence and factors associated with both underweight and stunting among Ugandan women aged 20 to 49 years. The prevalence of underweight and stunting were 6.9 and 1.3% respectively. Our study established that the factors associated with undernutrition among women aged 20 to 49 years were wealth index, residence and region. The prevalence of underweight was low compared to studies conducted in Nigeria (6.7%) [36] Kenya (9%) [37], and Tanzania (10%) [38]. This prevalence is also within the range of 5 to 20% reported for African women [36]. The observed differences in the underweight prevalence could be attributed to the differences in characteristics of study participants such as age as well as their food security status. In the study done in Nigeria by Senbanjo et al. only women aged 15–39 years from one state in Lagos were included while the other two studies from Tanzania and Kenya included women aged 15–49 years unlike our study that included women aged 20–49 years. In addition, Uganda having the lowest food insecurity in the East African region could also explain the lower underweight prevalence in Uganda compared to Tanzania and Kenya [39]. The prevalence of stunting is 1.3% almost similar to that in Kenya DHS (less than 1%) [37] and in Tanzania DHS (less than 3%) [38]. Underweight was significantly associated with residence, wealth index and region while stunting was only significantly associated with region.

Women who belonged to the Western, Eastern and Central regions were less likely to be underweight compared to women in the Northern region. Region of residence has also been shown to be associated with undernutrition in similar low-income African settings [20, 36, 37] and in Afghanistan [40]. The Northern region in Uganda is the most food insecure and the poorest [41]. This could be attributed to the fact that this region experienced a long civil war which greatly affected their agricultural production and the economy compared to the other regions that have been stable without civil conflicts [42]. The decreased agricultural production and poor economy which are majorly attributed to the civil war induced food insecurity by reducing own food production hence decreased food availability and access to food [43].. This leads to inadequate food in both quality and quantity risking them to underweight. Additionally, most people in the Northern region unlike the other regions are pastoral communities (some are nomadic) and this may negatively affect their consumption of the foods from agricultural origin (crops) as they mostly focus on mainly pastoral activities. As a matter of fact, pastoralism has been shown to increase risk of underweight in Ethiopian pastoral communities [20].

Women belonging to the poorest wealth quintiles were more likely to be underweight compared to those in the richest wealth quintiles. Wealth index/ socio-economic status has been shown to be significantly associated with underweight in other similar contexts [20, 30, 36]. Households that are facing poverty tend to have low purchasing power for food leading to both low quality and inadequate (quantity) food intake [20, 44]. The poor are usually less educated, and this negatively affects their nutritional knowledge. This limited knowledge on nutrition leads to inadequate dietary intake due to poor dietary consumption patterns like skipping meals and /or having unbalanced diets and eventually leading to underweight [4]. They also tend to have poor housing and inadequate access to clean water predisposing them to various infectious diseases such as diarrhea, tuberculosis which leads to loss of nutrients from the body hence leading to undernutrition [20, 45].

Our study also shows women who belonged to rural areas were 37% less likely to be underweight compared to their counterparts in urban areas. Although most studies in the region or within similar contexts show rural areas are prone to undernutrition [20, 46], a similar reverse association was observed in Bangladesh [47]. In Uganda, most agricultural production occurs in rural areas which contributes greatly to rural food availability and access [41]. Uganda is experiencing rapid urbanization that has led to the poorest and highly vulnerable people to settle in poorly organized informal urban areas [22, 48]**.** Despite the availability of regular food supply in urban areas, the urban poor households that make up the majority of the urban population have limited access to sufficient and nutritious food. This may compromise their ability to meet recommended dietary intake and therefore increasing their risk for underweight. This is supported by findings of a recent study that showed a high prevalence (88.5%) of food insecurity among women dwelling in one of the divisions of the most urbanized areas of the capital city Kampala [48]. The rapid increase in the urban settlements has also led to sanitation challenge and environmental degradation [22]. This has further led to people resorting to improvised unhygienic means of human excreta disposal that pose health risks such as diarrhea [12] that further increases the risk of underweight.

Stunting was only associated with region. Women in Central and Western regions were more likely to be stunted compared to those in the Northern region. This could be due to the intergenerational cycle of stunting where stunted children grow into stunted adolescents and adults. This is supported by the fact that according to the UDHS 2016 report and some studies that have looked at the trends of childhood stunting in Uganda since 1995, Western and Central regions have the highest prevalence [32, 45]. Furthermore, these studies have shown slow annual stunting reduction rate of 0.45% which increases the chances of these children growing into stunted adults and this could partly explain the high proportions and increased odds of stunting in western and central regions seen in this study. An alternative explanation to these findings could be the fact that Western Uganda is partly inhabited by the Batwa tribe, an indigenous pygmy population who are relatively short (in height) compared to other Ugandans [5, 16] and the possibility of migration of stunted women from other regions to the Central and Western regions which are the most economically vibrant regions.

### Strengths

We used a nationally representative sample and weighed the data for analysis and therefore our results are generalized to all Ugandan women aged 20 to 49 years. Secondly, we analyzed determinants of undernutrition considering contextual factors (household and community level factors) since also weighted data has been used. Thirdly, we used data with a large sample size which was collected, entered and cleaned by a team of trained and highly experienced scientists hence limiting mistakes in the data set.

### Limitations

The cross-sectional design is limited by lack of temporality hence causality inferences cannot be made. Most data on the predictors was based on self-reporting and could not be verified through records which risks socially acceptable answers hence information bias.

## Conclusion

Our study established that the factors associated with undernutrition among women aged 20 to 49 years were wealth index, residence and region. There is need to address socio-economic (contextual) factors mainly poverty and regional inequalities. There is also need for further studies to explain why stunting is highest among women with the highest wealth index.

## Supplementary Information

**Additional file 1: Figure 1.** Flow chat of sampling process. Figure 1 summarizes the selection and sampling process of study participants. **Table 5.** Background characteristics of 14,242 Ugandan women aged 20 to 49 years as per the 2016 UDHS. Shows the socio-demographic characteristics of all women aged 20 to 49 years including women who were not sampled for anthropometry.

## Data Availability

Access to the DHS data sets is openly available upon requests made to MEASURE DHS on their website (URL: https://www.dhsprogram.com/data/available-datasets.cfm).

## Abbreviations

EA: Enumeration area
AOR: Adjusted Odds Ratio
CI: Confidence Interval
COR: Crude Odds Ratio
DHS: Demographic Health Survey
UDHS: Uganda Demographic Health Survey
OR: Odds Ratio
SD: Standard Deviation
WHO: World Health Organization
BMI: Body Mass Index
GDP: Gross Domestic Product
SPSS: Statistical Package for Social Science
USAID: United States Agency for International Development
Kg: Kilograms
Cm: Centimetres
UNICEF: United Nations Children’s Fund
