# Supplementary material for: Factors associated with undernutrition among 20 to 49 year old women in Uganda: a secondary analysis of the Uganda demographic health survey 2016
Source: BMC Public Health. 2020 Nov 3;20:1644. doi: 10.1186/s12889-020-09775-2 (PMC7607648; doi:10.1186/s12889-020-09775-2)
Supplement: Supplementary file 1 — Additional file 1: Figure 1. Flow chat of sampling process. Figure 1 summarizes the selection and sampling process of study participants. Table 5. Background characteristics of 14,242 Ugandan women aged 20 to 49 years as per the 2016 UDHS. Shows the socio-demographic characteristics of all women aged 20 to 49 years including women who were not sampled for anthropometry. [file 12889_2020_9775_MOESM1_ESM.docx]

18,506 women aged 15 to 49 years

Excluded 4264 adolescents aged 15 to 19 years

14,242 women aged 20 to 49 years

9511 not sampled for anthropometry

4731 women sampled for anthropometry inclusion

91 Refused or not present for anthropometry

4640 women with anthropometry measurements

*Additional file Figure 1: flow chat of sampling process*

**Additional file table 5: Background characteristics of 14,242 Ugandan women aged 20 to 49 years as per the 2016 UDHS.**

| **Characteristics** | **N=14,242** | **%** |
| --- | --- | --- |
| Age |  |  |
| 20 to 29 | 6873 | 48.3 |
| 30 to 39 | 4554 | 32.0 |
| 40 to 49 | 2814 | 19.8 |
| **Residence** |  |  |
| Urban | 3909 | 27.4 |
| Rural | 10333 | 72.6 |
| **Region** |  |  |
| Western | 3618 | 25.4 |
| Eastern | 3631 | 25.5 |
| Central | 4349 | 30.5 |
| Northern | 2644 | 18.6 |
| **Sex household head** |  |  |
| Female | 4726 | 33.2 |
| Male | 9516 | 66.8 |
| **Household Size** |  |  |
| 6 and Above | 6763 | 47.5 |
| Less than 6 | 7479 | 52.5 |
| **Working status^a^** |  |  |
| Not working | 2780 | 19.5 |
| Working | 11462 | 80.5 |
| **Marital status** |  |  |
| Married | 10373 | 72.8 |
| Not married | 3869 | 27.2 |
| **Education Level** |  |  |
| No Education | 1705 | 12.0 |
| Primary Education | 7871 | 55.3 |
| Secondary Education | 3288 | 23.1 |
| Higher | 1378 | 09.7 |
| **Wealth Index** |  |  |
| Poorest | 2482 | 17.4 |
| Poorer | 2557 | 18.0 |
| Middle | 2644 | 18.6 |
| Richer | 2829 | 19.9 |
| Richest | 3730 | 26.2 |
